# Sickness Presence among Health Care Professionals: A Cross Sectional Study of Health Care Professionals in Slovenia

**DOI:** 10.3390/ijerph17010367

**Published:** 2020-01-06

**Authors:** Alenka Skerjanc, Metoda Dodic Fikfak

**Affiliations:** Clinical Institute of Occupational, Traffic and Sports Medicine, University Medical Centre Ljubljana, Zaloska Cesta 002, SI 1000 Ljubljana, Slovenia; metoda.dodicfikfak@kclj.si

**Keywords:** sickness presence, health care professionals, risk factors, interactions

## Abstract

Background and objectives: Presenteeism is a relatively new phenomenon that people, despite complaints and ill health that should prompt them to rest and take sick leave, go to work in any case. The highest sickness presence is largely to be found in the care and welfare and educational sectors. The aim of the study is to investigate the relations between different factors and sickness presence among health care professionals. Materials and Methods: A cross-sectional study was conducted at the largest hospital in Slovenia involving 5865 health care professionals employed at the University Medical Centre Ljubljana in the period between 1 January 2010 and 31 December 2010. Logistic regression methods were used to assess the associations between risk factors and their interactions and sickness presence. Results: Besides high odds for sickness presence in multivariate modelling for acute (OR = 359.7; 95%CI = 89.1–1452.8) and chronic disease (OR = 722.5; 95%CI = 178.5–2924.5) the highest odds were calculated for poor self-related health (OR = 3.0; 95%CI = 1.9–4.8), no possibility of replacement (OR = 1.9; 95%CI = 1.5–2.3), sickness absence > two times a year (OR = 1.6; 95%CI = 1.2–2.1), disabled workers (OR = 1.6; 95%CI = 1.0–2.5), and lower salary when on sick leave (OR = 1.5; 95%CI = 120–1.9). Risk factors interactions were not found to be associated with sickness presence among health care workers. Conclusions: The pre-requisite for higher sickness presence is workers’ bad health. The results indicate that sickness presence was associated with psycho social risk factors at work and their economic consequences. Continued sickness presence might have negative rather than positive consequences on work and health care professionals’ health in the future. Sickness presence needs to be taken into account for health care organizers.

## 1. Introduction

Presenteeism is a relatively new “phenomenon that people, despite complaints and ill health that should prompt them to rest and take sick leave, go to work in any case” [1,2]. It may facilitate rehabilitation and recovery, but it can exacerbate existing health problems and increase the risk of subsequent illness and absence as well as impair workability [3,4,5,6] and it can also endanger the clients’ and/or co-workers’ health [7]. Thirty to forty percent of employees are supposed not to have taken sick leave in the period of one year [8,9]. These are zero-absentees who are actually healthy and who do not experience health problems for a longer time as well as employees who feel sick, but work nevertheless. Those who are present at work despite sickness, therefore “sickness-present”, are supposed to have a lower social status, a fixed-term employment or are about to be laid off, can have more duties and obligations in the workplace, a high decision latitude, and they may have low possibility of replacement at work [1,2,5,9,10,11,12].

The highest sickness presence is largely to be found in the care and welfare and educational sectors [1,9]. Studies of sickness presence among health care professionals are conducted in Nordic countries [13,14], Great Britain [9], the Netherlands [10], the USA [7,15,16], and New Zealand [17]. Previous studies in individual groups of health care professionals have mostly investigated sickness presence associated with work-related factors. The level of sickness presence among health care professionals was associated with time pressure [9,10,13,14], shortage of personnel and low possibility of replacement [9,13], little work experience, and dissatisfaction with work [14]. In the investigated literature there is a shortage of studies to investigate possible association of life events, acute and chronic diseases, various health problems and disability, work impairment due to diseases or injuries with sickness presence among health care professionals, as well as age and gender differences [15,18,19]. This study includes all mentioned variables and their associations. It also includes the highest number of observed subjects ever.

This study is the first one that takes place in the Countries of Central and Eastern Europe (CCEE). The CCEE have similar health care systems and legislation. The legislation, Act on Health Care and Health Insurance in Slovenia gives a high level of security to worker once they get sick and take sick leave [20]. Namely payed sick leave duration has practically no time-limit if the disease requires sickness absence. Thus in this study we could preliminarily exclude the influence of pressure due to sick leave to presenteeism.

It was the aim of this study to research sickness presence among health care professionals in Slovenia which has not been done so far, and to test the relationships among different variables. The interactions between different variables and sickness presence among health care professionals were calculated. Finally, the models which explained sickness presence the best were created and used to evaluate the study.

## 2. Materials and Methods

Setting and population: The cross-sectional study was conducted at the largest hospital in Slovenia. The study population consisted of all heath care professionals (*N* = 5865) at the University Medical Centre Ljubljana (UMC Ljubljana) who were continuously employed there in the period between 1 January 2010 and 31 December 2010, one year prior to the data collection. All the employees who were on sick leave and/or maternity leave for more than six months in 2010 were excluded from the study.

Instrument: The questionnaire used in this study was an adaptation of questionnaires developed by Aronsson, Gustafsson, and Dallner [1], supplemented with some questions from Work Ability Index [21] and Holmes-Rahe Life Stress Inventory [22]. The questionnaire consisted of five sections: (1) Socio-demographic and life-style: sex, age, education, having children, smoking, recreation, and salary (9 questions). (2) Selected life events from Holmes-Rahe Stress Inventory: death of spouse or close family member, divorce, marriage, gain of a new family member, son or daughter leaving home, change in health of a family member, change to different type of work, change in responsibilities at work, change in work hours, additional education, change in financial state—partner’s loss of job, mortgage, change in living conditions, change in residence, change in recreation, vacation (16 questions). (3) Work related and psycho-social factors: workplace, tenure of employment, psychical and physical workload, shift work. (4) Organizational factors like devotedness to work, creativity, possibility of education, decision latitude, superiors’ and co-workers’ support, prolonged working hours, possibility of replacement and job satisfaction, lower salary when on sick leave (21 questions). (5) Health status of the participants: self-related health, disability, injuries, suffering from acute and chronic diseases, diagnoses and groups of diseases by organ systems (16 questions). (6) Work-ability: frequency of sick leave and work impairment due to diseases (10 questions).

They were open, multiple-choice, yes–no questions and items to be graded. Sickness presence is the study’s dependent variable and the participants were asked: “Has it happened over the previous 12 months that you have gone to work despite feeling that you really should have taken sick leave due to your state of health?” [2]. Responses were as by original authors dichotomized (0 = No, never/Yes, once, 1 = Yes, 2–5 times/Yes, more than 5 times). All employees who came to work at least twice or more times in 2010 when they felt sick were defined as sickness present, whereas those employees who were never sick and/or who came to work once at the most when they felt sick in 2010 were defined as non-sickness present.

Procedure: Data collection took place from February 2011 to May 2011, with one reminder. The questionnaires were distributed to all health care professionals in the UMC Ljubljana by the senior nursing officers of the departments and clinics in a closed envelope, accompanied by a covered letter explaining the purpose of the study and also envelope for the response. The questionnaires were completed independently and free and deposited in sealed boxes provided at each department or sent to the researchers by mail.

Statistical methods: Descriptive methods were used for data analysis to obtain indices of central tendency, variability, and dependence. In the second step univariate logistic regression was provided to determine the significance of single independent variable. Five logistic regression models were created in the third step to examine the associations between the significant independent variables and sickness presence. As explanatory variables we additionally created dummy variables (products among different independent variables for which we believed they interact with each other and thus explain sickness presence better than the single independent variable).

Five models were calculated: In the first model we included all variables related to life events which reached odds ratio (OR) and 95% confidence interval (CI) > 1 in previous univariate logistic regression and adjusted for demographic variables (Model 1); in the next model we included work related and psycho-social factors which reached OR and 95 % CI > 1 in previous univariate logistic regression and adjusted for demographic variables (Model 2); in Model 3 we included health measures which reached OR and 95 % CI > 1 in previous univariate logistic regression and adjusted for demographic variables ); then the full model with all significant variables from univariate logistic regression (Model 4); and finally models with dummy variables—interactions added to former models adjusted for demographic variables. Nagelkerke’s R squared and Hosmer Lemeshow Goodness-of-Fit Test were used to determine the fit of the models. The level of statistical significance was set at *p* < 0.05. Data processing was performed using SPSS version 20.0.

Ethical issues: The study was approved by the Ethical Committee of the Republic of Slovenia on the session 11.01.2011 (Document No. 118 and 119/01/11, dated 20.01.2011). The participants received verbal and written information on the study. Their participation was voluntary and informed consent was obtained. Confidentiality was guaranteed and data were analyzed in an aggregate from.

## 3. Results

The response rate for the present study was 57.8% (*N* = 3392). Thirteen questionnaires were excluded because they were incomplete. The response rate was higher for women than for men (*p* < 0.001). The age and educational level did not yield any statistically significant differences between respondents and non-respondents (*p* > 0.05), while the organizational units (*p* < 0.01) and occupation (*p* < 0.001) did. The highest response rate was among registered nurses (65.0%), the lowest among medical doctors (44.3%).

Among the respondents, 1927 (57.0%) declared that they were sickness present. 1446 (42.8%) 2–5 times, 481 (14.2%) six times or more, and 1452 (43.0%) that they were non-sickness present.

Among sickness present there were 1424 participants (42.1%) who stated that they were only sickness present and 503 participants (14.9%) who claimed they were sickness present and sickness absent. Among non-sickness present there were 1274 participants (37.7%) who answered that they were healthy in 2010 and 178 participants (5.3%) who reported that they were only sickness absent.

The study population included 2821 (83.5%) women and 558 (16.5%) men. The mean age was 41.6 years, there were 2557 (75.7%) participants less than 50 years old. There were 383 (11.4%) medical doctors, 2181 (64.5%) nurse and nurse auxiliaries, 371 (11.0%) other professionals within health care, 64 (1.9%) managers and 380 (11.2%) administrative workers. There were 1842 (54.5%) participants educated secondary school at the most, 1809 (53.5%) were married, 2428 (71.9%) with children, 868 (25.7%) smokers, 3065 (90.7%) recreationalists, less than 1000 EUR had 1715 (50.8%) participants. The greatest proportion of sickness present, 1301 (67.5%) belongs to nurses and nurse auxiliaries. Most commonly, sickness presence is observed in women aged less than 50, who finished primary or secondary school, whose net salary amount to less than 1000 EUR, with unfavorable life events (Table 1).

Among work-related and psychosocial characteristics there were statistically significant differences in sickness presence among those who work in irregular shifts which change quickly, have a lot of physical and psychical workload, who consider their work creative, who are supported by their superiors and/or co-workers, who experience time pressure at work (Table 2).

Health, self-reported health and workability presumed that sickness present healthcare professionals were more likely those with chronic diseases (Table 3).

In univariate analysis ORs for single demographic factors and life events ranged between 1.4 and 1.6. The disease of a relative (OR = 1.63, 95%CI = 1.27–2.09), partner′s loss of employment (OR = 1.42, 95%CI = 1.15–1.99) and mortgage (OR = 1.50, 95%CI = 1.27–1.78) were the highest. For factors associated with work and psycho-social factors ORs ranged between 1.6 and 2.3, the highest were time pressure (OR = 1.64, 95%CI = 1.42–1.90), shift work (OR = 1.81, 95%CI = 1.43–2.29), co-workers′ support (OR = 2.7, 95%CI = 1.57–2.54) and workplace satisfaction (OR = 2.25, 95%CI = 1.89–2.70). OR for diseases and problems related to workability ranged between 1.6 and 6.3, the highest were poor self-related health (OR = 6.30, 95%CI = 4.55–8.86), musculoskeletal disorders (OR = 2.61, 95%CI = 2.27–3.00) and disability (OR = 2.24, 95%CI = 1.75–3.40).

Finally we presented the model with all variables with OR and 95%CI > 1 in univariate analysis. This so called full model also fitted the best (Table 4).

Models with dummy variables, presenting the interactions among variables, were expected to add the significance to explain the sickness presence, but did not explain it better than Model 4, so they are not presented.

## 4. Discussion

This study is the first to the best of our knowledge to describe sickness presence in CCEE. Our findings indicate that the share of sickness present is comparable to the share mentioned in other studies [1,3,4,13,15]; however, the percentage of the employed without sick leave was greater than expected [8,9]. From the viewpoint of health protection, the proportion between sickness present and sickness absent is not favorable, since it forecasts the worsening of health of those who were sickness present [3,4]. The results suggest that the odds for sickness presence among health care professionals does not differ between both sexes, which is in line with the findings of one of the foreign studies [7], but the results of the studies carried out throughout the world differ [1,2,4,10]. As regards the age of sickness present, different studies yield different results [1,2,23]. The odds for sickness presence are more pronounced with workers aged less than 50, lower level of education and lower salary, which is also in accordance with the results of other studies [1,2,24,25].

For the first time, life events were systematically included in the study to test the association with sickness presence. The association between sickness presence and disease of a relative, partner’s loss of employment and the repayment of the mortgage on the one hand and stating that the salary is lower when on sick leave on the other show the economic dimensions of this phenomenon [1,2,11]. The financial distress is greater in groups with a lower level of education. Nevertheless, each occupational group has different reasons for a decision when sick leave is necessary and when to go to work despite health problems. But the greatest degree of sickness presence is noticed in employees who provide care or welfare services, or teach or instruct [1,25]. The previous studies assume that sickness presence is probably not caused by lower salary when on sick leave, but rather by the awareness that the work will have to be done by the colleagues, which imposes a greater burden on them [1,9,26]. The risk for sickness presence can be observed when time pressure is present, when there is low possibility of replacement, when work cannot be done by someone else, when some obligations cannot be postponed [1,2,9,17,18,27]. Tenure of employment in UMC Ljubljana less than 15 years may be associated with sickness presence because younger workers lack experiences to organize themselves at work and they are not skilled enough to predict obstacles and solutions for problems at work to carry out all the work properly in selected time schedule [2,18,27,28,29].

The perception of presence of acute or chronic diseases is the logical crucial prerequisite for sickness presence that aggravates normal work and reduces the availability for work. Besides describing the association of sickness presence with acute and chronic disease [15,18], our study also aims to describe the weight of evidence of other variables. We assume that they reflect the current socio-economic relations in the society. Among diseases associated with sickness presence, respiratory diseases occupy the first place. In hospitals more than 70% of employees reported that they had been at work while sick with acute respiratory disease [7]. Employees who report the relationships to their colleagues as trustworthy and satisfactory tended to report a higher rate of sickness presence [29] and the consequences for the patients, as well as for the colleagues, may be unfavorable in these cases [7,16]. Among the psycho-social risk factors for sickness presence in health care professionals there is low of possibility of replacement at the first place. It is this distress that can exert negative influence on mental health and trigger a vicious circle that leads to weariness and exhaustion [30,31,32]. So far sickness presence caused by economic factors and also by responsibility and devotion to one’s work and co-workers often has negative rather than positive consequences on work and worker’s health [3,7,33]. A sick person cannot do the work in the same way as a healthy one, the consequences being mistakes at work and more time spent on the performance of work tasks in comparison with a healthy worker [34]. Mental and behavioral disorders such as mild depression, tension, anxiety, insomnia, fatigue, and exhaustion also occupy the high place among the groups of diagnoses linked with sickness presence [35,36]. We think that also a certain burden of musculoskeletal disorders, gastrointestinal diseases, and cardiovascular diseases triggered by psychosomatic mechanisms are later presented as the formal reason for sick leave instead of mental health disorders and a positive association between sickness absence and sickness presence was also confirmed in this study [3,4]. Disabled health care professionals who suffer from invalidity and have the degree of their disability formally recognized by the law [37] should have their workplaces adapted regarding their limitations. As disabled workers report that they are more often at work when they are sick, investigations should be performed if their workplaces are adjusted to their psychical and physical abilities. Namely, work related factors can be even more important than personal factors when deciding for sickness absence or sickness presence [17,18,26].

We supposed that sickness presence is not associated only with separate factors. We also supposed that different factors, when interacting, could produce higher and different effects compared with separate, independent effects, but we could not prove it. Despite the fact that to our knowledge this is the first known study to take them into account our calculations did not prove the importance of possible interaction effect. If the independent variables entering the models had been weak, then the interactions might have risen their impact. There are studies claiming that the individuals are not passive; they make a conscious choice of whether or not to attend work [19,29]. This choice is articulated within a system of social and economic pressures, as well as contextual factors such as legislative and compensation environment [19,38]. The current availability of workers to provide their job is dependent on their perception and determination of their functional capabilities and education, knowledge, age, and motivation. There are also current personal and life conditions which are very important issue such as health or disease, children, partner’s employment status, income, workplace demands like time pressure, superior- and co-workers’ relations, and the possibility of work adaption or replacement. For the same worker, the interactions of the same variables can be different in different situations. This non-differentiated exposure misclassification should be studied in future.

Despite limitations such as possible recall bias and robust measures there are also preferences like the high number of participants. Although suggestive, our findings are not proof of causation. Further studies are needed mostly to research the late consequences of sickness presence.

## 5. Conclusions

The pre-requisite for higher sickness presence is workers’ bad health. The results indicate that sickness presence was associated with psycho social risk factors at work and their economic consequences. If continued sickness presence might have negative rather than positive consequences on work and health worker’s health in the future, it needs to be taken into account by health care organizers.

## Figures and Tables

**Table 1:** Socio-demographic characteristics and life events of the study subjects.

Characteristics	Sickness Present (*N* = 1927)	Non-Sickness Present (*N* = 1452)
Sex [N (%)] *		
Female	1634 (84.8)	1187 (81.7)
Male	293 (15.2)	265 (18.3)
Age group [N (%)] *		
<50 Years	1510 (78.4)	1047 (72.1)
≥50 Years	417 (21.6)	405 (27.9)
Education [N (%)] *		
Secondary School Education at the Most	1130 (58.6)	712 (49.0)
More than Secondary School	797 (41.4)	740(51.0)
Children [N (%)] *		
Yes	1417 (73.5)	1011 (69.6)
No	510 (26.5)	441(30.4)
Smoker [N (%)] *		
Yes	554 (28.7)	314 (21.6)
No	1373 (71.3)	1138 (78.4)
Recreational [N (%)]*		
Yes	1721 (89.3)	1344 (92.6)
No	206 (10.7)	108 (7.4)
Net salary [N (%)] *		
<1000 EUR	1030 (53.5)	685 (47.2)
≥1000 EUR	897 (46.5)	767 (52.8)
Death of Spouse or Close Family Member [N (%)] *		
Yes	456 (23.7)	290 (2.0)
No	1471 (76.3)	1162 (98.0)
Disease of A Relative [N (%)] *		
Yes	205 (10.6)	99 (6.8)
No	1722 (89.4)	1353(93.2)
Partner’s Loss of Employment [N (%)] *		
Yes	164 (8.5)	84 (5.8)
No	1763 (91.5)	1368 (94.2)
Mortgage [N (%)] *		
Yes	470 (24.4)	257 (17.7)
No	1457 (75.6)	1195 (82.3)

* *p* < 0.05.

**Table 2:** Work-related and psychosocial characteristics of the study subjects.

Characteristics	Sickness Present (*N* = 1927)	Non-Sickness Present (*N* = 1452)
Work [N (%)] *		
Medical Doctor	221 (11.5)	162 (11.2)
Nurses and Nurse Auxiliary	1301 (67.5)	880 (60.6)
Other Professionals within Health Care	177 (9.2)	194 (13.4)
Managers	37 (1.9)	27 (1.9)
Administrative Workers	191 (9.9)	189 (13.0)
Tenure of Employment in UMC > 15 Years [N (%)] *		
Yes	1003 (52.0)	819 (56.4)
No	924 (48.0)	633 (43.6)
High Psychical Workload [N (%) *]		
Yes	1233 (64.0)	754 (51.9)
No	694 (36.0)	698 (48.1)
High Physical Workload [N (%)] *		
Yes	672 (34.9)	351 (24.2)
No	1255 (65.1)	1101 (75.8)
Irregular Shifts [N (%)] *		
Yes	249 (12.9)	110 (7.6)
No	1678 (87.1)	1342 (92.4)
Creative Work [N (%)] *		
Yes	1544 (80.1)	1265 (87.1)
No	383 (19.9)	187 (12.9)
Possibility of Education [N (%)] *		
Yes	1308 (67.9)	1137 (78.3)
No	619 (32.1)	315 (21.7)
High Decision Latitude [N (%)] *		
Yes	921 (47.8)	860 (59.2)
No	1006 (52.2)	592 (40.8)
Superiors’ Support [N (%)] *		
Yes	1361 (70.6)	1209 (83.3)
No	566 (29.4)	243 (16.7)
Co-workers’ Support [N (%)] *		
Yes	1671 (86.7)	1336 (92.0)
No	256 (13.3)	116 (8.0)
Time Pressure [N (%)] *		
Yes	801 (41.6)	439 (30.2)
No	1126 (58.4)	1013 (69.8)
Satisfaction in the Workplace [N (%)] *		
Yes	1408 (73.1)	1248 (86.0)
No	519 (26.9)	204 (14.0)
Possibility of Replacement [N(%)] *		
Yes	673 (34.9)	980 (67.5)
No	1254 (65.1)	472 (32.5)
Lower Salary when on Sick Leave [N (%)] *		
Yes	779 (40.4)	224 (15.4)
No	1148 (59.6)	1228 (84.6)

* *p* < 0.05.

**Table 3:** Health status and work ability of the study subjects.

Characteristics	Sickness Present (*N* = 1927)	Non-Sickness Present (*N* = 1452)
Poor Self-Related Health [N (%)] *		
Yes	300 (15.6)	41 (2.8)
No	1627 (84.4)	1411 (97.2)
Disabled [N (%)] *		
Yes	183 (9.5)	65 (4.5)
No	1744 (90.5)	1387 (95.5)
Musculoskeletal Disorders [N (%)] *		
Yes	1143 (59.3)	521 (35.9)
No	784 (40.7)	931 (64.1)
Cardiovascular Diseases [N (%)] *		
Yes	465 (24.1)	228 (15.7)
No	1462 (75.9)	1224 (84.3)
Respiratory Diseases [N (%)] *		
Yes	492 (25.5)	165 (11.4)
No	1435 (74.5)	1287 (88.6)
Mental and Behavioral Disorders [N (%)] *		
Yes	1284 (66.6)	388 (26.7)
No	643 (33.4)	1064 (73.3)
Acute Disease in 2010 [N (%)] *		
Yes	1349 (70.0)	616 (42.4)
No	578 (30.0)	836 (57.6)
Chronic Disease in 2010 [N (%)] *		
Yes	1501 (77.9)	292 (20.1)
No	426 (22.1)	1160 (79.9)
Sickness Absent ≥ 2 Times [N (%)] *		
Yes	503 (26.1)	178 (12.3)
No	1424 (73.9)	1274 (87.7)

* *p* < 0.05.

**Table 4:** Logistic regression models with odds ratios (ORs) and their 95 confidence interval (CI) for associations between sickness presence and life events (Model 1), work related and psycho-social factors (Model 2), health measures (Model 3), and the full model (Model 4).

Independent Variable	Model 1 OR (95%CI)	*p*	Model 2 OR (95%CI)	*p*	Model 3 OR (95%CI)	*p*	Model 4 OR (95%CI)	*p*
Sex								
Female/Male	1.13 (0.94–1.37)	0.203	1.41 (1.14–1.74)	0.001	0.81 (0.63–1.05)	0.115	1.04 (0.78–1.38)	0.812
Age Group								
<50 Years/≥50 Years	1.49 (1.26–1.77)	0.000	1.28 (1.06–1.54)	0.009	1.56 (1.22–2.00	0.000	1.32 (1.00–1.74)	0.049
Education								
Secondary School Education at the Most/More than Secondary School	1.35 (1.11–1.64)	0.003	1.39 (1.12–1.72)	0.003	1.12 (0.86–1.45)	0.413	1.11 (0.82–1.49)	0.509
Children								
Yes/No	1.27 (1.07–1.49)	0.005	1.48 (1.24–1.77)	0.000	1.21 (0.97–1.51)	0.097	1.49 (1.14–1.94)	0.003
Smoker								
Yes/No	1.31 (1.11–1.55)	0.001	1.37 (1.15–1.63)	0.000	1.32 (1.05–1.66)	0.018	1.36 (1.07–1.74)	0.012
Recreationalist								
Yes/No	0.70 (0.55–0.91)	0.006	0.83 (0.64–1.07)	0.154	0.81 (0.58–1.15)	0.237	0.86 (0.60–1.23)	0.408
Net Salary								
<1000 EUR/≥1000 EUR	0.98 (0.81–1.18)	0.818	1.07 (0.87–1.32)	0.506	1.03 (0.79–1.33)	0.833	1.06 (0.79–1.42)	0.698
Death of Spouse or Close Family Member								
Yes/No	1.15 (0.97–1.36)	0.118					0.95 (0.74–1.21)	0.665
Disease of a Relative								
Yes/No	1.51 (1.17–1.96)	0.002					1.16 (0.82–1.65)	0.410
Partner’s Loss of Employment								
Yes/No	1.35 (1.02–1.79)	0.035					0.84 (0.57–1.23)	0.360
Mortgage								
Yes/No	1.38 (1.15–1.65)	0.000					1.06 (0.82–1.34)	0.648
Work								
Nurses and Nurse Auxiliary/Medical Doctor			0.74 (0.56–0.98)	0.035			0.57 (0.39–0.83)	0.004
Other Professionals within Health Care/Medical Doctor			0.67 (0.481–0.93)	0.017			0.53 (0.37–0.83)	0.006
Managerial Worker/Medical Doctor			0.83 (0.47–1.48)	0.534			0.72 (0.32–1.64)	0.002
Administrative Worker/Medical Doctor			0.52 (0.37–0.75)	0.000			0.45 (0.28–0.74)	0.002
Tenure of employment in UMC > 15 years								
No/Yes			1.30 (1.10–1.55)	0.003			1.57 (1.22–2.03)	0.001
High Psychical Workload								
Yes/No			1.41 (1.20–1.66)	0.000			1.02 (0.81–1.27)	0.893
High Physical Workload								
Yes/No			1.22 (1.03–1.46)	0.025			1.11 (0.86–1.42)	0.893
Superiors’ Support								
Yes/No			1.31 (1.54–2.16)	0.010			0.90 (0.72–1.13)	0.179
Co-workers’ Support								
Yes/No			1.19 (0.92–1.53)	0.196			0.88 (0.61–1.26)	0.472
Time Pressure								
Yes/No			1.83 (1.54–2.16)	0.000			1.47 (1.17–1.86)	0.001
Satisfaction in the Workplace								
Yes/No			1.45 (1.18–1.79)	0.000			1.11 (0.83–1.51)	0.001
Possibility of Replacement								
No/Yes							1.89 (1.52–2.34)	0.000
Lower salary when on Sick Leave								
Yes/No							1.48 (1.16–1.90)	0.002
Poor Self Related Health								
Yes/No					3.59 (2.30–5.60)	0.000	2.98 (1.87–4.76)	0.000
Disabled								
Yes/No					1.83 (1.18–2.84)	0.007	1.57 (1.00–2.49)	0.054
Musculoskeletal Disorders								
Yes/No					1.16 (0.94–1.43)	0.181	1.07 (0.86–1.34)	0.549
Cardiovascular Diseases								
Yes/No					1.09 (0.84–1.41)	0.523		
Respiratory Diseases								
Yes/No					1.42 (1.09–1.85)	0.010	1.34 (1.02–1.76)	0.037
Mental and Behavioral Disorders								
Yes/No					1.32 (1.06–1.64)	0.014	1.06 (0.84–1.34)	0.619
Acute Disease								
Yes/No					457.29 (113.59–1840.96)	0.000	359.72 (89.06–1452.84)	0.000
Chronic Disease								
Yes/No					886.94 (219.66–3581.26)	0.000	722.52 (178.50–2924.48)	0.000
Sickness Absent ≥ 2 Times								
Yes/No							1.58 (1.21–2.06)	0.001
Nagelkerke R^2^	0.06		0.30		0.64		0.67	
Hosmer Lemershow Test	0.78		0.17		0.15		0.27	
% Variability Explained	60.0		72.9		79.3		81.3	

Models 1–4 are adjusted for demographic variables.
